# Asthma Cured by External Application

**Published:** 1850-01

**Authors:** Wm. Townsend

**Affiliations:** Royal Centre, Indiana


### ARTICLE VI.

*Asthma Cured by External Applications.*—By Dr. Wm. Townsend, of Royal Centre, Indiana.

Messrs. Editors :—

In looking over my diary, I find the following cases of Asthma :

1. Mrs. —, aged 25 years:—had suffered at times for seven years. After using, at different times, all the remedies laid down by Drs. Eberle and Watson, some of which proved palliative, but not curative, I purged well with Senna and Salts, and applied over the lower part of the breast, a plaster made of portions of Burgundy Pitch and Rosin, with sufficient tallow to bring it to a consistency to spread. This was sprinkled with Pulv. Opii and Gum Camphor, and was worn for thre weeks, and it is now worn two or three days every four weeks. Two years have now passed, and there have been none of the symptoms of the Asthma, since the first application of the plaster.

Case 2. Mrs. – aged 44 yeas: Had suffered for 4 years, at intervals. On the 4th of March last, I was called in haste to see her. I found her suffering beyond description, and from Idiosyncracy, opium could not be used. I directly had a poultice made of corn meal, and applied over the breast and stomach, which, after being renewed two or three times, gave relief. I then purged with Senna and Salts. On the 5th, I found her with some of the symptoms of Asthma, and some suffering. I applied the above plaster, and kept her bowels in motion with Senna. She wears the plaster as the former patient did, and has had none of the symptoms since the first application.

